# Altered cholesterol homeostasis in critical illness-induced muscle weakness: effect of exogenous 3-hydroxybutyrate

**DOI:** 10.1186/s13054-021-03688-1

**Published:** 2021-07-17

**Authors:** Chloë Goossens, Ruben Weckx, Sarah Derde, Sarah Vander Perre, Inge Derese, Paul P. Van Veldhoven, Bart Ghesquière, Greet Van den Berghe, Lies Langouche

**Affiliations:** 1 Clinical Division and Laboratory of Intensive Care Medicine, Department of Cellular and Molecular Medicine, KU Leuven, Herestraat 49 bus 503, 3000 Leuven, Belgium; 2 Laboratory for Lipid Biochemistry and Protein Interactions, Department of Cellular and Molecular Medicine, KU Leuven, 3000 Leuven, Belgium; 3 Metabolomics Expertise Center, Center for Cancer Biology, VIB, KU Leuven, 3000 Leuven, Belgium

**Keywords:** Sepsis, Muscle weakness, Cholesterol, Ketone

## Abstract

**Background:**

Muscle weakness is a complication of critical illness which hampers recovery. In critically ill mice, supplementation with the ketone body 3-hydroxybutyrate has been shown to improve muscle force and to normalize illness-induced hypocholesterolemia. We hypothesized that altered cholesterol homeostasis is involved in development of critical illness-induced muscle weakness and that this pathway can be affected by 3-hydroxybutyrate.

**Methods:**

In both human critically ill patients and septic mice, the association between circulating cholesterol concentrations and muscle weakness was assessed. In septic mice, the impact of 3-hydroxybutyrate supplementation on cholesterol homeostasis was evaluated with use of tracer technology and through analysis of markers of cholesterol metabolism and downstream pathways.

**Results:**

Serum cholesterol concentrations were lower in weak than in non-weak critically ill patients, and in multivariable analysis adjusting for baseline risk factors, serum cholesterol was inversely correlated with weakness. In septic mice, plasma cholesterol correlated positively with muscle force. In septic mice, exogenous 3-hydroxybutyrate increased plasma cholesterol and altered cholesterol homeostasis, by normalization of plasma mevalonate and elevation of muscular, but not hepatic, expression of cholesterol synthesis genes. In septic mice, tracer technology revealed that 3-hydroxybutyrate was preferentially taken up by muscle and metabolized into cholesterol precursor mevalonate, rather than TCA metabolites. The 3-hydroxybutyrate protection against weakness was not related to ubiquinone or downstream myofiber mitochondrial function, whereas cholesterol content in myofibers was increased.

**Conclusions:**

These findings point to a role for low cholesterol in critical illness-induced muscle weakness and to a protective mechanism-of-action for 3-hydroxybutyrate supplementation.

**Supplementary Information:**

The online version contains supplementary material available at 10.1186/s13054-021-03688-1.

## Introduction

Limb and respiratory muscle weakness develop in the majority of critically ill patients who require a prolonged stay in the intensive care unit (ICU), referred to as ICU-acquired weakness. The pathophysiology of ICU-acquired weakness is complex and multifactorial with underlying mechanisms that negatively affect muscle function independently from loss of muscle mass [1]. This debilitating complication is associated with greater post-ICU functional impairment, prolonged hospitalization, delayed rehabilitation and late death. Effective therapies, other than avoiding modifiable risk factors, are currently lacking [1–4].

Recently, we have shown in critically ill mice that supplementation with 3-hydroxybutyrate (3HB) protects against muscle weakness [5]. In this sepsis-induced mouse model of critical illness, 3HB supplementation did not affect the illness-induced loss of muscle mass, whereas it did attenuate illness-induced loss of muscle force. Key underlying mechanisms of muscle weakness, such as atrophy, autophagy and inflammation, were not affected by 3HB and 3HB also did not appear to serve as an alternative energy substrate [5]. Remarkably, 3HB treatment increased plasma LDL cholesterol up to normal levels [5]. This was a surprising observation, as critical illness in humans and in mice is typically characterized by a robust, immediate and sustained decrease in plasma cholesterol concentrations (LDL and HDL), which is proportional to the severity of illness and the risk of death [6–9]. Ketone bodies can theoretically serve as precursors of cholesterol, after conversion into acetyl-CoA. Indeed, earlier studies that mainly focused on brain and liver have shown that ketone bodies, under specific circumstances such as during development and starvation, can be a preferred substrate for cholesterogenesis [10–15].

There is emerging evidence also pointing to a role for cholesterol in controlling muscle function. For example, cholesterol-lowering statin therapy can evoke muscle weakness and aches [16]. Such side effects of statins have been linked to impaired mitochondrial function caused by reduced ubiquinone levels, a derivative of the cholesterol precursor mevalonate and an essential co-factor in the mitochondrial respiratory chain [16, 17]. In skeletal muscle, cholesterol also plays an important role in the regulation of myofiber membrane fluidity and signal transduction processes. Depletion of myofiber membrane cholesterol has been shown to hamper muscle contractions [18–20], whereas hereditary muscular dystrophy is associated with increased myofiber membrane cholesterol [21, 22]. Despite emerging evidence highlighting a role of cholesterol in controlling muscle function and the known hypocholesterolemia of critical illness, the link between low cholesterol and ICU-acquired weakness has not been investigated.

We hypothesized that altered cholesterol homeostasis plays a role in the development of ICU-acquired weakness and that the protective effect of 3HB supplementation on weakness is related to its effects on cholesterol homeostasis. These hypotheses were tested in a human study and in a clinically relevant and validated mouse model of critical illness evoked by sepsis.

## Methods

### Patient study—experimental setup, methods and statistical analysis

Patients were enrolled in the EPaNIC study, a randomized controlled study that compared providing early parenteral nutrition (early-PN) with allowing a macronutrient deficit during the first 7 days in ICU (late-PN) [23]. In a subset of 600 patients, muscle strength was quantified with the MRC sum score on post-randomization day 8 ± 1 [23, 24]. Cholesterol concentrations were measured with the Amplex™ Red Cholesterol Assay Kit (Thermo Fisher Scientific, Waltham, MA, USA) in stored serum samples taken on admission and day 3 (or the last day if patients had a shorter ICU stay). Multivariable regression analysis was used to assess association between serum cholesterol and muscle strength, after adjustment for study randomization and baseline characteristics [age, gender, body mass index (BMI), nutritional risk score (NRS), severity of illness (APACHE II), diabetes, malignancy, pre-admission dialysis, sepsis upon admission and admission diagnostic categories] and treatment with statin.

### Mouse studies—experimental setup, methods and statistical analysis

Male 24-week-old C57BL/6J mice were anaesthetized and received a catheter in the jugular vein, sepsis was induced by ligation and puncture of the cecum and mice were treated with antibiotics and analgesia twice daily. After surgery, mice received continuous fluid resuscitation with a mixture of balanced colloids and crystalloids for 20 h, thereafter continuous intravenous infusion of standard mixed PN (Olimel N7E, Baxter, Lessines, Belgium) at 5.8 kcal/day [5, 25].

In the first animal study, mice received twice daily subcutaneous injections of either 150 mg/day D,L-3HB sodium salt or isocaloric/isovolumetric D-glucose as placebo. Pair-fed healthy mice served as controls. After 5 days, mice were anesthetized and sacrificed by cardiac puncture, plasma, liver and skeletal muscle were snap-frozen. Gene expression analyses were performed as previously described [26] with commercial TaqMan® assays (Applied Biosystems, Additional file 1: Table S1). Plasma cholesterol was measured with the Amplex™ Red Cholesterol Assay Kit, plasma mevalonate and ubiquinone-9 concentrations were measured with LC/MS by BioNotus. Muscle citrate synthase and mitochondrial respiratory chain complex activities were measured with spectrophotometry as described previously [27]. For the quantification of muscle cholesterol content, lipids were extracted [28] and total cholesterol was measured with the Amplex™ Red Cholesterol Assay Kit.

In the second animal study, on day 2 or day 5, septic mice received a subcutaneous injection of 75 mg D,L-3HB-^13^C_4_ sodium salt (606030, Sigma Aldrich, St. Louis, MO, USA). As controls, full-fed mice received an equal tracer bolus injection. Two hours post-injection, mice were anaesthetized and sacrificed by cardiac puncture, plasma, liver and skeletal muscle were snap-frozen. Tracer incorporation analysis was performed using a Dionex UltiMate 3000 LC System in-line connected to a Q-Exactive Orbitrap mass spectrometer.

Normally distributed data were compared with Student’s t tests or one-way analysis of variance (ANOVA) with post hoc Fisher's LSD test for multiple comparisons of Student’s t tests, where necessary after transformation to obtain a near-normal distribution (JMP®Pro12, SAS Institute Inc). Not-normally distributed data were analyzed with nonparametric Wilcoxon or Median tests. Two-sided *p* values ≤ 0.05 were considered statistically significant in all analyses.

Additional detail on the mouse models and methods is provided in Additional file 1.

## Results

### The development of muscle weakness in critically ill patients is associated with low serum cholesterol concentrations

In 600 human critically ill patients, we first investigated whether serum cholesterol concentrations were associated with the development of muscle weakness (Table 1). Muscle weakness was assessed in these patients 1 week after admission to the ICU, either in the ICU or on the regular ward, as part of a prospectively planned secondary analysis of the EPaNIC RCT, which compared 2 nutritional strategies [23, 24]. We quantified serum total cholesterol concentration on the ICU admission day and on day 3 or last ICU day (LD) for those patients who stayed in ICU for less than 3 days. Critically ill patients who developed muscle weakness displayed lower serum cholesterol concentrations than non-weak patients both on admission (mean ± SE 67.9 ± 2.5 mg/dl vs. 79.4 ± 2.0 mg/dl; *p* = 0004) and on day 3/LD (mean ± SE 67.6 ± 2.1 mg/dl vs. 77.8 ± 1.8 mg/dl; *p* = 0.0002). In multivariable analysis adjusted for the randomized nutritional intervention and for baseline risk factors, low day 3/LD cholesterol concentrations remained independently associated with the subsequent development of muscle weakness (Table 2). Of the 600 studied patients, 141 (23.5%) received statin therapy in the ICU at the time of cholesterol measurement on day 3/LD. As compared with patients who did not receive statins, those who did displayed lower serum cholesterol (mean ± SE 66.9 ± 2.1 mg/dl vs. 76.1 ± 1.3 mg/dl; univariate *p* = 0.01, multivariate *p* = 0.04, Additional file 1: Table S2). Statin therapy itself, however, did not associate with the occurrence of weakness (*p* = 0.2). Adding statin therapy to the multivariable model further strengthened the association between low serum cholesterol and subsequent development of weakness (Table 2).

**Table 1:** Baseline characteristics of weak and non-weak critically ill patients

	Weak patients^A^ (*n* = 232)	Non-weak patients^A^ (*n* = 368)	*p* value
Age—median (IQR)	64.3 (55.8–73.1)	62.2 (50.7–73.3)	0.01
Male gender—*n* (%)	132 (56.9)	221 (60.1)	0.4
BMI (kg/m^2^)—median (IQR)	24.8 (22.1–29.1)	25.2 (22.9–28.4)	0.3
NRS ≥ 5—*n* (%)	95 (40.9)	82 (22.3)	< 0.0001
APACHEII—median (IQR)	35 (29–40)	26 (18–34)	< 0.0001
Diabetes—*n* (%)	37 (15.9)	59 (16.0)	1
Malignancy—*n* (%)	67 (28.9)	96 (26.1)	0.4
Pre-admission dialysis—*n* (%)	4 (1.7)	2 (0.5)	0.2
Sepsis upon admission—*n* (%)	141 (60.8)	123 (33.4)	< 0.0001
ICU admission categories—*n* (%)			< 0.0001
Surgical ICU, emergency	100 (43.1)	159 (43.2)	
Cardiac elective surgery	63 (27.2)	139 (37.8)	
Other elective surgery	8 (3.4)	27 (7.3)	
Medical ICU	61 (26.3)	43 (11.7)	
Randomized to early-PN—*n* (%)	127 (54.7)	168 (45.6)	0.03

^A^ Muscle weakness was assessed by the Medical Research Center (MRC) sum score: a score of less than 48 is considered weak [24]. BMI is body mass index, or weight in kilograms divided by the square of the height in meters. APACHEII reflects scores on the Acute Physiology and Chronic Health Evaluation II (APACHE II) range from 0 to 71, with higher scores indicating a greater severity of illness. NRS reflects Nutritional Risk Screening (NRS) scores which range from 0 to 7, with higher scores indicating a higher risk of malnutrition

**Table 2:** Multivariate analysis determining the impact of day 3/LD serum cholesterol on the development of ICU-acquired muscle weakness in critically ill patients

Acquisition of ICU-acquired weakness^A^		
	Odd ratio (95% CI)	*p* value
Role of serum cholesterol		
Serum cholesterol day 3/LD (per mg/l added)	0.94 (0.89–1.00)	0.05
Randomization to late-PN^B^	0.69 (0.47–1.00)	0.05
Baseline risk factors		
Age (per year added)	1.01 (1.00–1.03)	0.005
Male gender	0.84 (0.57–1.24)	0.4
BMI (25–40 kg/m^2^)	0.83 (0.55–1.23)	0.3
NRS ≥ 5	1.41 (0.92–2.16)	0.1
APACHE II (per unit added)	1.10 (1.07–1.13)	< 0.0001
Presence of diabetes	0.72 (0.41–1.23)	0.2
Presence of malignancy	0.90 (0.57–1.42)	0.6
Pre-admission dialysis	2.15 (0.35–13.29)	0.3
Sepsis upon admission	2.26 (1.42–3.59)	0.0005
Admission categories (compared with medical ICU)		
Surgical ICU, emergency	0.68 (0.39–1.16)	0.1
Surgical ICU, elective	1.83 (0.61–5.45)	0.2
Cardiac surgery	1.84 (0.90–3.75)	0.09
Impact of statin therapy		
Statin therapy	0.66 (0.40–1.08)	0.1
Serum cholesterol day 3/LD (per mg/l added)	0.94 (0.89–0.99)	0.03
Randomization to late-PN^B^	0.68 (0.46–0.99)	0.04
Baseline risk factors		
Age (per year added)	1.02 (1.00–1.03)	0.002
Male gender	0.86 (0.58–1.27)	0.4
BMI (25–40 kg/m^2^)	0.86 (0.58–1.29)	0.4
NRS ≥ 5	1.45 (0.94–2.23)	0.08
APACHE II (per unit added)	1.10 (1.07–1.13)	< 0.0001
Presence of diabetes	0.79 (0.45–1.37)	0.4
Presence of malignancy	0.89 (0.57–1.41)	0.6
Pre-admission dialysis	2.06 (0.33–12.58)	0.4
Sepsis upon admission	0.44 (0.28–0.70)	0.0006
Admission categories (compared with medical ICU)		
Surgical ICU, emergency	0.71 (0.41–1.23)	0.2
Surgical ICU, elective	1.83 (0.61–5.46)	0.2
Cardiac surgery	2.04 (1.00–4.20)	0.05

^A^Muscle weakness was assessed by the Medical Research Center (MRC) sum score: a score of less than 48 is considered weak [24].

^B^The EPaNIC RCT showed that withholding parenteral nutrition until beyond the first week in intensive care unit (ICU) (late-PN) was clinically superior to early parenteral nutrition supplementing insufficient enteral nutrition (early-PN): it shortened ICU stay, quickened recovery and reduced the acquisition of ICU-acquired weakness [23, 24]. Study randomization did not affect day 3/LD cholesterol concentrations (72.7 ± 2.0 mg/dl in early-PN patients vs. 74.9 ± 2.0 mg/dl in late-PN patients; *p* = 0.2). See also Additional file 1: Table S2 for the independent association between the factors in this model and serum cholesterol day 3/LD. BMI is body mass index, or weight in kilograms divided by the square of the height in meters. APACHEII reflects scores on the Acute Physiology and Chronic Health Evaluation II (APACHE II) range from 0 to 71, with higher scores indicating a greater severity of illness. NRS reflects Nutritional Risk Screening (NRS) scores which range from 0 to 7, with higher scores indicating a higher risk of malnutrition

### In septic mice, low plasma cholesterol concentrations correlated with reduced muscle force and both were increased with 3HB supplementation

**Fig. 1:**
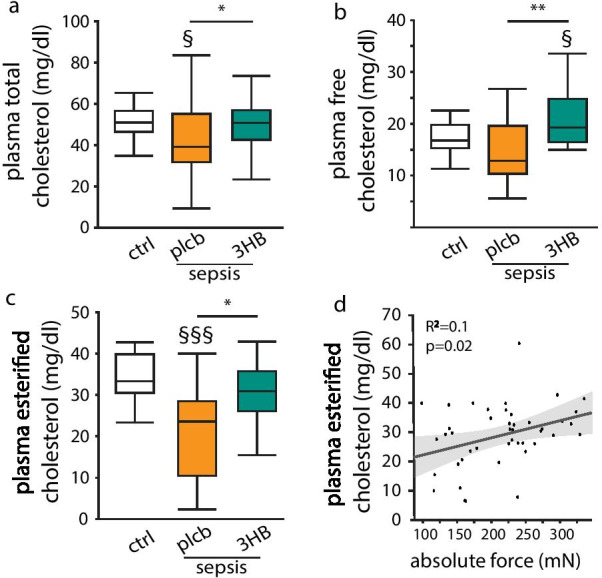
Effect of 3-hydroxybutyrate on plasma cholesterol concentrations and correlations with muscle force. Septic mice received parenteral nutrition supplemented with 3-hydroxybutyrate (3HB) or isocaloric glucose as placebo (plcb). **a** Plasma total cholesterol concentrations. **b** Plasma free cholesterol concentrations. **c** Plasma esterified cholesterol concentrations. **d** Correlation between absolute maximal muscle force and plasma esterified cholesterol concentrations. Gray line and spread represent fit. Absolute maximal muscle force was previously quantified in the extensor digitorum longus (EDL) muscle with ex vivo muscle force measurements [5] [*White:* healthy control (ctrl) mice, *n* = 15; *orange*: septic mice receiving placebo, *n* = 17; *green*: septic mice receiving 3HB, *n* = 17]. Data in boxplots represent median and interquartile range; dots represent outliers. §*p* ≤ 0.05, §§*p* ≤ 0.001 between healthy controls and septic mice, *p ≤ 0.05, **p ≤ 0.01, between groups of septic mice

In parenterally fed prolonged septic mice, plasma total cholesterol concentrations were decreased after 5 days of illness as compared with healthy controls (Fig. 1a). This was mostly due to reduced plasma concentrations of esterified cholesterol, not free cholesterol (Fig. 1b, c). Total, free and esterified plasma cholesterol concentrations were increased by 3HB supplementation as compared with placebo (isocaloric glucose), up to levels of healthy control mice (Fig. 1a–c). Absolute muscle force correlated positively with plasma esterified cholesterol concentrations (*R*^2^ = 0.1, *p* = 0.02) (Fig. 1d), whereas no clear association was observed with plasma free cholesterol (*R*^2^ = 0.009, *p* = 0.5). The observed differences between placebo-treated and 3HB-treated mice were not due to a difference in survival (17/20 (85%) for the placebo group and 17/21 (82%) for the 3HB group, *p* = 0.8, Additional file 1: Fig. S1).

### In septic mice, 3HB supplementation altered cholesterol homeostasis in skeletal muscle, not in liver

**Fig. 2:**
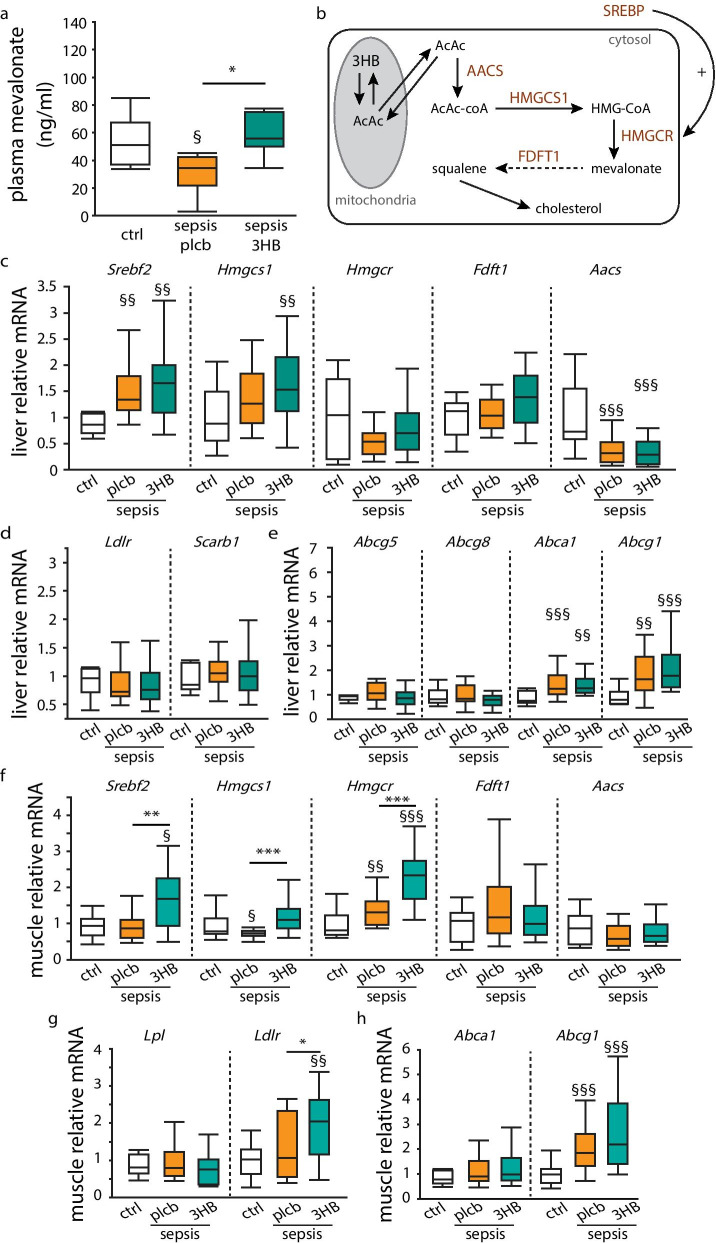
Effect of 3-hydroxybutyrate supplementation on cholesterol homeostasis. Septic mice received parenteral nutrition supplemented with 3-hydroxybutyrate (3HB) or isocaloric glucose as placebo (plcb). **a** Plasma mevalonate concentrations. **b** Overview of main enzymes involved in cholesterogenesis. **c** Relative mRNA levels of genes involved in hepatic cholesterol synthesis, **d** hepatic cholesterol uptake, **e** hepatic cholesterol efflux, **f** muscle cholesterol synthesis, **g** muscle cholesterol uptake and **h** muscle cholesterol efflux. Gene expression data are normalized to *Hprt* or *Rn18s* and shown relative to the mean of healthy controls (ctrl) [*White:* healthy controls, *n* = 15; *orange*: septic mice receiving placebo, *n* = 17; *green*: septic mice receiving 3HB, *n* = 17]. Data in boxplots represent median and interquartile range; dots represent outliers. §*p* ≤ 0.05 §§*p* ≤ 0.01 §§§*p* ≤ 0.001 between healthy controls and septic mice, *p ≤ 0.05, **p ≤ 0.01, ***p ≤ 0.001 between groups of septic mice

Given that 3HB supplementation in septic mice increased plasma cholesterol concentrations, we further investigated the effect of 3HB on cholesterol homeostasis during sepsis. Plasma mevalonate, a cholesterol precursor of which plasma concentrations reflect cholesterol synthesis [29, 30], was lower in placebo-treated septic mice than in healthy mice and was normalized with 3HB supplementation (Fig. 2a).

As the liver is the major site for cholesterogenesis and for production of esterified cholesterol to be used by peripheral tissues, we first assessed hepatic gene expression of key markers of hepatic cholesterol synthesis, uptake and release (Fig. 2b). Hepatic gene expression of the main regulator of the cholesterol pathway, *Srebf2*, was increased by sepsis whereas hepatic cholesterol-synthesizing enzymes *Hmgcs1* and *Fdft1* were unaffected, and *Aacs* expression was decreased compared to healthy mice—all irrespective of 3HB treatment (Fig. 2c). *Hmgcr* expression was upregulated in 3HB treated mice only (Fig. 2c). Hepatic expression of LDL-cholesterol uptake receptor *Ldlr* and HDL-cholesterol uptake receptor *Scarb1* was unaffected by sepsis or 3HB supplementation (Fig. 2d). Similarly, hepatic expression of transporters involved in export of cholesterol to bile and gut *(Abcg5* and *Abcg8*) was unaffected by sepsis or 3HB supplementation (Fig. 2e). In contrast, hepatic expression of transporters of cholesterol to the circulation was upregulated by sepsis, but again not further affected by 3HB supplementation (Fig. 2e).

Since de novo cholesterol synthesis can occur in most cell types, and 3HB did not appear to directly affect hepatic cholesterol homeostasis in septic mice, we also assessed whether 3HB could have an effect on cholesterol homeostasis in skeletal muscle. In contrast to liver, in muscle tissue, expression of *Srebf2* was increased by 3HB supplementation (Fig. 2f). Supplementation of 3HB in septic mice also caused a normalization of *Hmgcs1* and elevated *Hmgcr* compared with both placebo-treated septic mice and with healthy mice, whereas *Fdft1* and *Aacs* were neither affected by illness nor by 3HB supplementation (Fig. 2f). LDL-cholesterol receptor *Ldlr* was higher in septic mice receiving 3HB compared to placebo-treated septic mice and healthy mice, whereas *Lpl* expression was unaffected (Fig. 2g). Expression of cholesterol efflux transporter *Abca1* was unaffected by illness and of *Abcg1* increased with sepsis (Fig. 2h).

**Fig. 3:**
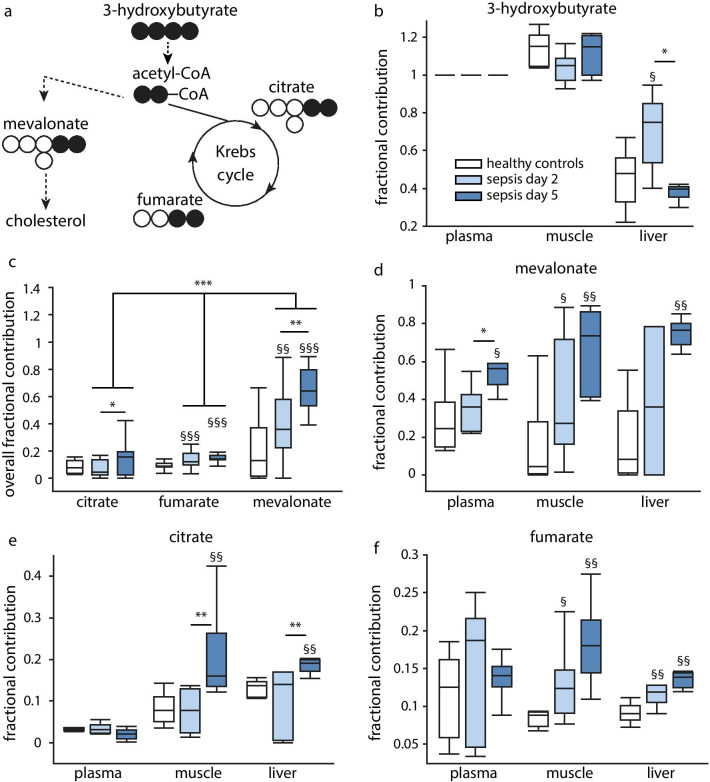
The fate of supplemented 3-hydroxybutyrate as cholesterogenic or TCA cycle substrate. **a** Tracer conversion overview. For each individual mouse, the fractional contribution of each labeled metabolite (indicating the fraction labeled metabolite of the total amount of metabolite) in each tissue was corrected for the fractional contribution of 3HB in plasma. **b** Tissue-specific uptake of ^13^C-labeled 3-hydroxybutyrate. **c** Overall fractional contribution of metabolites. Tissue-specific fractional labeling of **d** mevalonate, **e** citrate and **f** fumarate [*White:* healthy controls *n* = 7, *light blue:* septic day 2 mice *n* = 7, *dark blue:* septic day 5 mice *n* = 7]. Data in boxplots represent median and interquartile range; dots represent outliers. §*p* ≤ 0.05 §§*p* ≤ 0.01 §§§*p* ≤ 0.001 between healthy controls and septic mice, *p ≤ 0.05, **p ≤ 0.01, ***p ≤ 0.001 between day 2 and day 5 of sepsis

To study ketone-to-cholesterol metabolism in more detail, we performed a second mouse study using tracer technology to investigate the fate of supplemented ^13^C_4_-3-hydroxybutyrate (^13^C-3HB) as cholesterogenic or tricarboxylic acid (TCA) cycle substrate in acute (day 2) and prolonged (day 5) sepsis-induced critically ill mice (Fig. 3a). At tissue level, supplemented ^13^C-3HB was more abundantly present in skeletal muscle than in plasma and less present in liver (Fig. 3b). In general, mevalonate was the preferred metabolite of injected ^13^C-3HB during sepsis, with several-fold higher conversion of ^13^C-3HB to ^13^C-mevalonate than to TCA cycle metabolites citrate and fumarate on both day 2 and day 5 in septic mice, but not in healthy mice (Fig. 3c). Compared to healthy mice, ^13^C-mevalonate was sixfold (day 2) and 16-fold (day 5) increased in skeletal muscle, whereas in plasma and liver, ^13^C-mevalonate content was only elevated on day 5 of sepsis (Fig. 3d). In plasma, metabolites ^13^C-citrate and ^13^C-fumarate were not affected by sepsis, whereas the fractional contribution of both metabolites was generally similarly increased by illness in skeletal muscle and liver, but to a much lower extent than mevalonate (Fig. 3e, f).

### In septic mice, 3HB supplementation-induced altered cholesterol homeostasis did not affect muscle function via ubiquinone, but did affect myofiber cholesterol content

**Fig. 4:**
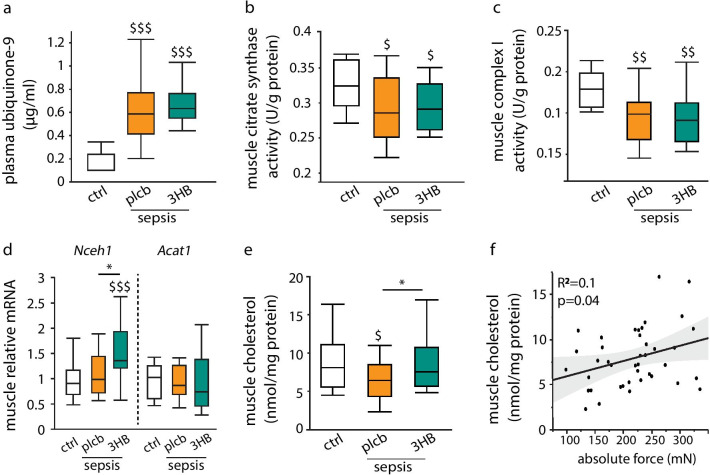
Effect of 3-hydroxybutyrate supplementation on plasma ubiquinone, myofiber mitochondrial function and myofiber cholesterol content. Septic mice received parenteral nutrition supplemented with 3-hydroxybutyrate (3HB) or isocaloric glucose as placebo (plcb). **a** Plasma ubiquinone-9 concentration [*White:* healthy controls (ctrl), *n* = 13; *orange*: septic mice receiving placebo, *n* = 15; *green*: septic mice receiving 3HB, *n* = 14]. **b** Mitochondrial citrate synthase and **c** complex I activities per gram protein in skeletal muscle. **d** Muscle gene expression analysis of enzymes involved in cholesterol esterification. Data are normalized to *Rn18s* and shown relative to the mean of controls. **e** Myofiber cholesterol content per mg protein in skeletal muscle. **f** Correlation between absolute maximal muscle force and muscle cholesterol content. Gray line and spread represent fit. Absolute maximal muscle force was previously quantified in the extensor digitorum longus (EDL) muscle with ex vivo muscle force measurements [5] [*White:* healthy controls, *n* = 15; *orange*: septic mice receiving placebo, n = 17; *green*: septic mice receiving 3HB, *n* = 17]. Data in boxplots represent median and interquartile range; dots represent outliers. §*p* ≤ 0.05 §§*p* ≤ 0.01 §§§*p* ≤ 0.001 between healthy controls and septic mice, *p ≤ 0.05, **p ≤ 0.01, ***p ≤ 0.001 between groups of septic mice

As 3HB appeared to be preferentially shuttled into the cholesterol pathway in skeletal muscle during sepsis, and improved muscle function has been observed in 3HB-supplemented septic mice, we also investigated pathways that link cholesterol to muscle function in septic mice. The most direct pathway is via the mevalonate-derived mitochondrial co-factor ubiquinone, which is essential for mitochondrial electron transport chain function. However, both in placebo-treated and 3HB-treated septic mice, plasma ubiquinone concentrations were equally higher than in healthy mice (Fig. 4a). Furthermore, reduced activities of mitochondrial citrate synthase and complex I, indicative of mitochondrial dysfunction, were observed in skeletal muscle of both placebo-treated and 3HB-treated septic mice (Fig. 4b, c). This suggests that the observed effects of 3HB on both cholesterol and muscle function were unrelated to ubiquinone and downstream mitochondrial function.

Besides its key role in mitochondrial function, myofiber cholesterol content is also essential for normal muscle contractility. Most myofiber cholesterol resides within the cell membranes as free cholesterol. After uptake or intracellular synthesis, esterified cholesterol is hydrolyzed to free cholesterol by NCEH1 before being transported to the membranes. In septic mice, 3HB caused an elevation in *Nceh1* expression in skeletal muscle, compared to both placebo-treated septic mice and healthy mice, whereas expression of the main enzyme for cholesterol esterification, *Acat1*, was unaffected by sepsis and 3HB (Fig. 4d). Furthermore, myofiber cholesterol content was decreased in septic mice receiving placebo, but upregulated to normal levels in 3HB-treated mice (Fig. 4e). In addition, absolute muscle force significantly correlated with the myofiber cholesterol content (Fig. 4f), whereas this was not the case for citrate synthase activity (*R*^2^ = 0.02, *p* = 0.3), or complex I activity (*R*^2^ = 0.01, *p* = 0.4).

## Discussion

We here demonstrated that altered cholesterol homeostasis is involved in the development of critical illness-induced muscle weakness, in both human critically ill patients and septic mice. Furthermore, the previously observed muscle protection of 3HB supplementation during sepsis could be linked to effects on cholesterol homeostasis. In mice, critical illness-induced hypocholesterolemia was prevented with 3HB supplementation. Supplemented 3HB was preferentially taken up by skeletal muscle and used as substrates for cholesterogenesis. Not ubiquinone formation with downstream muscular mitochondrial function, but myofiber cholesterol content was increased and associated with muscle force.

Acute and sustained low circulating cholesterol concentrations are a hallmark of critical illness and considered a marker of poor prognosis [6–9]. The acute decrease in cholesterol has been interpreted as linked to increased cortisol production and to endotoxin-scavenging functions, but the exact pathophysiology remains unclarified [31–35]. We demonstrated in critically ill patients that reduced circulating cholesterol levels were associated with the subsequent development of muscle weakness, and this independently from the severity or type of critical illness and independently from whether patients received early parenteral nutrition. Also in septic mice, the low plasma cholesterol concentrations and the low myofiber cholesterol content correlated with impaired muscle force generation. In addition, 3HB supplementation of septic mice was able to normalize cholesterol levels in plasma and in skeletal muscle and its protective effects on muscle force could be linked to increased myofiber cholesterol content.

Statin-induced lowering of cholesterol has been linked to statin-induced muscle aches and muscle weakness [16]. In this context, research has mainly focused on ubiquinone, a derivative of the cholesterol precursor mevalonate, which is an essential co-factor in the mitochondrial respiratory chain [16, 17]. Ubiquinone deficiency and impaired mitochondrial function have been described during sepsis [36, 37]. Plasma ubiquinone is often used as clinical proxy for functional tissue levels [34]. Remarkably, we found increased plasma ubiquinone concentrations in all septic mice, unaffected by 3HB supplementation. Possibly, these high plasma levels are due to leaking of ubiquinone into the circulation after sepsis-induced mitochondrial and/or tissue damage. Mitochondrial function was indeed found to be impaired in skeletal muscle of the septic mice, but it was unaffected by 3HB supplementation and mitochondrial functional test results did not correlate with muscle force. In contrast, total cholesterol content in myofibers was lowered by sepsis, increased by 3HB supplementation and positively correlated with muscle force. Furthermore, increased gene expression of *Nceh1* in muscle may suggest enhanced hydrolysis of de novo synthetized cholesterol, and such hydrolyzed cholesterol is the main form of cholesterol present in cell membranes. Depletion of myofiber membrane cholesterol has indeed been shown to impair muscle contraction [18–20].

In mice, 3HB supplementation not only increased cholesterol content in myofibers, also plasma cholesterol concentration was increased. However, how 3HB supplementation affected plasma cholesterol concentrations remains speculative. Hepatic cholesterol homeostasis was largely unaffected by 3HB supplementation, suggesting that the liver was not involved in altered plasma cholesterol. Possibly, increased uptake of 3HB in skeletal muscle, followed by ketone-to-cholesterol metabolism, may have reduced the need for muscular cholesterol uptake from the circulation. Whether this was sufficient to increase plasma cholesterol levels, or whether other mechanisms in other organs may be involved remains to be investigated further. In general, high-fat ketogenic diets tend to increase circulating cholesterol concentrations, possibly through higher consumption of dietary cholesterol in combination with enhanced ketone-to-cholesterol metabolism [38, 39]. Conversely, exogenous administration of ketone esters has been shown to either not affect [40] or reduce [41] plasma cholesterol concentrations. However, the experiments with ketone esters were performed in healthy full-fed rodents and humans, which is quite a different context than that of critical illness which is hallmarked by low circulating cholesterol. Additionally, in healthy individuals, ketone bodies are primarily shuttled into the TCA cycle, which could theoretically reduce the availability of acetyl-CoA for cholesterogenesis [42]. In septic mice however, it has already been observed that ketone bodies are preferential used as signaling molecules, not energy substrates, indicating that other metabolic pathways may be activated [5]. Indeed, with state-of-the art tracer technology we here could demonstrate that conversion of 3HB to mevalonate was several-fold higher than conversion to the TCA cycle metabolites citrate and malate in septic animals, though not in healthy mice.

Whether direct cholesterol substitution therapy could benefit the critically ill patient has not been investigated. Therapies that mimic the endotoxin-scavenging role of cholesterol, such as treatment with a phospholipid emulsion or polymyxin B hemoperfusion, have been unsuccessful in improving outcome [43, 44]. At present, a Phase I/II feasibility trial is ongoing to test whether cholesterol levels can be stabilized with a lipid emulsion in septic patients, but no clinical endpoints are investigated yet [45]. Therefore, the novel finding presented here, an effect of 3HB supplementation on cholesterol homeostasis during sepsis, is quite promising. In our mice experiments, 3HB did not cause the hepatic adversities associated with infusion of high lipid doses [5], and the local effect of 3HB on cholesterol homeostasis in skeletal muscle may be an advantage. In addition, 3HB supplementation in septic mice has previously been shown to stimulate markers of muscle regeneration, a process that is impaired in patients suffering from ICU-acquired weakness [5, 46]. This dual effect of 3HB on muscle force generation and muscle regeneration may support the therapeutic potential, which should be further investigated.

This study also has limitations. First, we used a multivariate logistic regression model to assess the independent association of plasma cholesterol with weakness in human critically ill patients. Due to the inherent limitations of such a statistical analysis, we cannot exclude that the observed effect of cholesterol on muscle weakness is partly confounded by other factors. The presence of sepsis was the strongest determinant for muscle weakness and also is a strong suppressor of plasma cholesterol [9]. However, the independent association, observed in our controlled septic animal model, strongly argues against this confounder. Second, mice typically have much lower plasma LDL cholesterol and higher plasma HDL compared to humans [47]. We assessed total plasma cholesterol in both the human and animal study, but extrapolations on the contribution of the different lipoproteins between these species have to be done with caution. Future studies on the role of cholesterol in sepsis should consider using humanized mice models to better mimic the human setting [48, 49].

## Conclusions

This study describes a possible role of cholesterol in preventing ICU-acquired weakness and points toward a mechanism-of-action underlying the 3HB-induced protection against muscle weakness in sepsis. These findings open novel perspectives for prevention and/or treatment of this debilitating complication of critical illness.

## Supplementary Information

**Additional file 1.Supplementary method section; Table S1:** List of commercial TaqMan® assays; **Table S2:** Multivariate linear regression analysis of factors determining day 3/LD serum cholesterol; **Figure S1** – Survival analysis of 3HB vs. placebo treated septic mice.

## Data Availability

Some or all datasets generated during and/or analyzed during the current study are not publicly available but are available from the corresponding author on reasonable request.

## Abbreviations

3HB: 3-Hydroxybutyrate
BMI: Body mass index
EDL: Extensor digitorum longus
ICU: Intensive care unit
LD: Last day
NRS: Nutritional risk score
PN: Parenteral nutrition
TCA: Tricarboxylic acid

## References

[1] Vanhorebeek I, Latronico N, Van den Berghe G (2020). ICU-acquired weakness. Intensive Care Med.

[2] Hermans G, Van den Berghe G (2015). Clinical review: intensive care unit acquired weakness. Crit Care.

[3] Hermans G, Van Mechelen H, Clerckx B, Vanhullebusch T, Mesotten D, Wilmer A, Casaer MP, Meersseman P, Debaveye Y, Van Cromphaut S (2014). Acute outcomes and 1-year mortality of intensive care unit-acquired weakness. A cohort study and propensity-matched analysis. Am J Respir Crit Care Med.

[4] Fletcher SN, Kennedy DD, Ghosh IR, Misra VP, Kiff K, Coakley JH, Hinds CJ (2003). Persistent neuromuscular and neurophysiologic abnormalities in long-term survivors of prolonged critical illness. Crit Care Med.

[5] Goossens C, Weckx R, Derde S, Dufour T, Vander Perre S, Pauwels L, Thiessen SE, Van Veldhoven PP, Van den Berghe G, Langouche L (2019). Adipose tissue protects against sepsis-induced muscle weakness in mice: from lipolysis to ketones. Crit Care.

[6] Mesotten D, Swinnen JV, Vanderhoydonc F, Wouters PJ, Van den Berghe G (2004). Contribution of circulating lipids to the improved outcome of critical illness by glycemic control with intensive insulin therapy. J Clin Endocrinol Metab.

[7] Gordon BR, Parker TS, Levine DM, Saal SD, Wang JC, Sloan BJ, Barie PS, Rubin AL (2001). Relationship of hypolipidemia to cytokine concentrations and outcomes in critically ill surgical patients. Crit Care Med.

[8] Dunham CM, Fealk MH, Sever WE (2003). Following severe injury, hypocholesterolemia improves with convalescence but persists with organ failure or onset of infection. Crit Care.

[9] Tanaka S, Labreuche J, Drumez E, Harrois A, Hamada S, Vigue B, Couret D, Duranteau J, Meilhac O (2017). Low HDL levels in sepsis versus trauma patients in intensive care unit. Ann Intensive Care.

[10] Hasegawa S, Noda K, Maeda A, Matsuoka M, Yamasaki M, Fukui T (2012). Acetoacetyl-CoA synthetase, a ketone body-utilizing enzyme, is controlled by SREBP-2 and affects serum cholesterol levels. Mol Genet Metab.

[11] Koper JW, Lopes-Cardozo M, Van Golde LM (1981). Preferential utilization of ketone bodies for the synthesis of myelin cholesterol in vivo. Biochim Biophys Acta.

[12] Geelen MJ, Lopes-Cardozo M, Edmond J (1983). Acetoacetate: a major substrate for the synthesis of cholesterol and fatty acids by isolated rat hepatocytes. FEBS Lett.

[13] Lopes-Cardozo M, Klein W (1985). Contribution of acetoacetate to the synthesis of cholesterol and fatty acids in regions of developing rat brain in vivo. Neurochem Int.

[14] Patel MS, Johnson CA, Rajan R, Owen OE (1975). The metabolism of ketone bodies in developing human brain: development of ketone-body-utilizing enzymes and ketone bodies as precursors for lipid synthesis. J Neurochem.

[15] Robinson AM, Williamson DH (1980). Physiological roles of ketone-bodies as substrates and signals in mammalian-tissues. Physiol Rev.

[16] Stroes ES, Thompson PD, Corsini A, Vladutiu GD, Raal FJ, Ray KK, Roden M, Stein E, Tokgozoglu L, Nordestgaard BG (2015). Statin-associated muscle symptoms: impact on statin therapy-European Atherosclerosis Society Consensus Panel Statement on Assessment, Aetiology and Management. Eur Heart J.

[17] Qu H, Guo M, Chai H, Wang WT, Gao ZY, Shi DZ (2018). Effects of coenzyme Q10 on statin-induced myopathy: an updated meta-analysis of randomized controlled trials. J Am Heart Assoc.

[18] Barrientos G, Llanos P, Hidalgo J, Bolanos P, Caputo C, Riquelme A, Sanchez G, Quest AF, Hidalgo C (2015). Cholesterol removal from adult skeletal muscle impairs excitation-contraction coupling and aging reduces caveolin-3 and alters the expression of other triadic proteins. Front Physiol.

[19] Vega-Moreno J, Tirado-Cortes A, Alvarez R, Irles C, Mas-Oliva J, Ortega A (2012). Cholesterol depletion uncouples beta-dystroglycans from discrete sarcolemmal domains, reducing the mechanical activity of skeletal muscle. Cell Physiol Biochem.

[20] Launikonis BS, Stephenson DG (2001). Effects of membrane cholesterol manipulation on excitation-contraction coupling in skeletal muscle of the toad. J Physiol.

[21] de Kretser TA, Livett BG (1977). Skeletal-muscle sarcolemma from normal and dystrophic mice. Isolation, characterization and lipid composition. Biochem J.

[22] Srivastava NK, Yadav R, Mukherjee S, Pal L, Sinha N (2017). Abnormal lipid metabolism in skeletal muscle tissue of patients with muscular dystrophy: In vitro, high-resolution NMR spectroscopy based observation in early phase of the disease. Magn Reson Imaging.

[23] Casaer MP, Mesotten D, Hermans G, Wouters PJ, Schetz M, Meyfroidt G, Van Cromphaut S, Ingels C, Meersseman P, Muller J (2011). Early versus late parenteral nutrition in critically ill adults. N Engl J Med.

[24] Hermans G, Casaer MP, Clerckx B, Guiza F, Vanhullebusch T, Derde S, Meersseman P, Derese I, Mesotten D, Wouters PJ (2013). Effect of tolerating macronutrient deficit on the development of intensive-care unit acquired weakness: a subanalysis of the EPaNIC trial. Lancet Respir Med.

[25] Derde S, Thiessen S, Goossens C, Dufour T, Van den Berghe G, Langouche L. Use of a central venous line for fluids, drugs and nutrient administration in a mouse model of critical illness. Jove-J Vis Exp. 2017;123:e55553. 10.3791/55553 PMC5565154 28518095

[26] Langouche L, Marques MB, Ingels C, Gunst J, Derde S, Vander Perre S, D'Hoore A, Van den Berghe G (2011). Critical illness induces alternative activation of M2 macrophages in adipose tissue. Crit Care.

[27] Thiessen SE, Derese I, Derde S, Dufour T, Pauwels L, Bekhuis Y, Pintelon I, Martinet W, Van den Berghe G, Vanhorebeek I (2017). The role of autophagy in critical illness-induced liver damage. Sci Rep.

[28] Van Veldhoven PP, Meyhi E, Mannaerts GP (1998). Enzymatic quantitation of cholesterol esters in lipid extracts. Anal Biochem.

[29] Waldron J, Webster C (2011). Liquid chromatography-tandem mass spectrometry method for the measurement of serum mevalonic acid: a novel marker of hydroxymethylglutaryl coenzyme A reductase inhibition by statins. Ann Clin Biochem.

[30] Parker TS, McNamara DJ, Brown CD, Kolb R, Ahrens EH, Alberts AW, Tobert J, Chen J, De Schepper PJ (1984). Plasma mevalonate as a measure of cholesterol synthesis in man. J Clin Invest.

[31] Levels JH, Abraham PR, van den Ende A, van Deventer SJ (2001). Distribution and kinetics of lipoprotein-bound endotoxin. Infect Immun.

[32] Bhakdi S, Tranum-Jensen J, Utermann G, Fussle R (1983). Binding and partial inactivation of Staphylococcus aureus alpha-toxin by human plasma low density lipoprotein. J Biol Chem.

[33] Parker TS, Levine DM, Chang JC, Laxer J, Coffin CC, Rubin AL (1995). Reconstituted high-density lipoprotein neutralizes gram-negative bacterial lipopolysaccharides in human whole blood. Infect Immun.

[34] Marik PE. Adrenal insufficiency: the link between low apolipoprotein A-I levels and poor outcome in the critically ill? Crit Care Med. 2004; 32(9):1977–1978; author reply 1978–1979. 10.1097/01.ccm.0000132895.89019.32 15343040

[35] van der Voort PHJ, Gerritsen RT, Bakker AJ, Boerma EC, Kuiper MA, de Heide L (2003). HDL-cholesterol level and cortisol response to synacthen in critically ill patients. Intensive Care Med.

[36] Donnino MW, Cocchi MN, Salciccioli JD, Kim D, Naini AB, Buettner C, Akuthota P (2011). Coenzyme Q10 levels are low and may be associated with the inflammatory cascade in septic shock. Crit Care.

[37] Brealey D, Brand M, Hargreaves I, Heales S, Land J, Smolenski R, Davies NA, Cooper CE, Singer M (2002). Association between mitochondrial dysfunction and severity and outcome of septic shock. Lancet.

[38] Kwiterovich PO, Vining EP, Pyzik P, Skolasky R, Freeman JM (2003). Effect of a high-fat ketogenic diet on plasma levels of lipids, lipoproteins, and apolipoproteins in children. JAMA.

[39] Creighton BC, Hyde PN, Maresh CM, Kraemer WJ, Phinney SD, Volek JS (2018). Paradox of hypercholesterolaemia in highly trained, keto-adapted athletes. BMJ Open Sport Exerc Med.

[40] Kesl SL, Poff AM, Ward NP, Fiorelli TN, Ari C, Van Putten AJ, Sherwood JW, Arnold P, D'Agostino DP (2016). Effects of exogenous ketone supplementation on blood ketone, glucose, triglyceride, and lipoprotein levels in Sprague-Dawley rats. Nutr Metab (Lond).

[41] Kemper MF, Srivastava S, Todd King M, Clarke K, Veech RL, Pawlosky RJ (2015). An ester of beta-hydroxybutyrate regulates cholesterol biosynthesis in rats and a cholesterol biomarker in humans. Lipids.

[42] Webber RJ, Edmond J (1977). Utilization of L(+)-3-hydroxybutyrate, D(-)-3-hydroxybutyrate, acetoacetate, and glucose for respiration and lipid synthesis in the 18-day-old rat. J Biol Chem.

[43] Dellinger RP, Bagshaw SM, Antonelli M, Foster DM, Klein DJ, Marshall JC, Palevsky PM, Weisberg LS, Schorr CA, Trzeciak S (2018). Effect of targeted polymyxin B hemoperfusion on 28-day mortality in patients with septic shock and elevated endotoxin level: the EUPHRATES randomized clinical trial. JAMA.

[44] Dellinger RP, Tomayko JF, Angus DC, Opal S, Cupo MA, McDermott S, Ducher A, Calandra T, Cohen J, Lipid I (2009). Efficacy and safety of a phospholipid emulsion (GR270773) in Gram-negative severe sepsis: results of a phase II multicenter, randomized, placebo-controlled, dose-finding clinical trial. Crit Care Med.

[45] Guirgis FW, Black LP, Rosenthal MD, Henson M, Ferreira J, Leeuwenburgh C, Kalynych C, Moldawer LL, Miller T, Jones L, et al. LIPid Intensive Drug therapy for Sepsis Pilot (LIPIDS-P): Phase I/II clinical trial protocol of lipid emulsion therapy for stabilising cholesterol levels in sepsis and septic shock. BMJ Open 2019; 9(9):e029348. 10.1136/bmjopen-2019-029348 PMC6756323 31537565

[46] Dos Santos C, Hussain SN, Mathur S, Picard M, Herridge M, Correa J, Bain A, Guo Y, Advani A, Advani SL (2016). Mechanisms of chronic muscle wasting and dysfunction after an intensive care unit stay. A pilot study. Am J Respir Crit Care Med.

[47] Yin W, Carballo-Jane E, McLaren DG, Mendoza VH, Gagen K, Geoghagen NS, McNamara LA, Gorski JN, Eiermann GJ, Petrov A (2012). Plasma lipid profiling across species for the identification of optimal animal models of human dyslipidemia. J Lipid Res.

[48] Trinder M, Wang Y, Madsen CM, Ponomarev T, Bohunek L, Daisely BA, Julia Kong H, Blauw LL, Nordestgaard BG, Tybjaerg-Hansen A (2021). Inhibition of cholesteryl ester transfer protein preserves high-density lipoprotein cholesterol and improves survival in sepsis. Circulation.

[49] Oppi S, Luscher TF, Stein S (2019). Mouse models for atherosclerosis research-which is my line?. Front Cardiovasc Med.

