# Supplementary material for: Altered cholesterol homeostasis in critical illness-induced muscle weakness: effect of exogenous 3-hydroxybutyrate
Source: Crit Care. 2021 Jul 17;25:252. doi: 10.1186/s13054-021-03688-1 (PMC8285799; doi:10.1186/s13054-021-03688-1)
Supplement: Supplementary file 1 — Additional file 1.Supplementary method section; Table S1: List of commercial TaqMan® assays; Table S2: Multivariate linear regression analysis of factors determining day 3/LD serum cholesterol; Figure S1 – Survival analysis of 3HB vs. placebo treated septic mice. [file 13054_2021_3688_MOESM1_ESM.docx]

**Altered cholesterol homeostasis in critical illness-induced muscle weakness:**

**effect of exogenous 3-hydroxybutyrate**

**Authors**: Chloë Goossens, Ruben Weckx, Sarah Derde, Sarah Vander Perre, Inge Derese, Paul P Van Veldhoven, Bart Ghesquière, Greet Van den Berghe and Lies Langouche

Online additional method section

Additional file 1: Table S1: List of commercial TaqMan® assays

Additional file 1: Table S2: Multivariate linear regression analysis

Additional file 1: figure S1: Survival analysis of 3HB vs. placebo treated septic mice

**Supplementary method section**

***Mouse studies – experimental setup***

Male 24 week old C57BL/6J mice were anaesthetized and received a catheter in the jugular vein, sepsis was induced by ligation and puncture of the cecum and mice were treated with antibiotics and analgesia twice daily. Six hours post-operatively, and hereafter every 12 hours until sacrifice, subcutaneous antibiotics and analgesia were given. Mice were excluded if catheter-related problems occurred. After surgery, mice received continuous fluid resuscitation with a mixture of balanced colloids and crystalloids for 20 hours, thereafter continuous intravenous infusion of standard mixed PN (Olimel N7E, Baxter, Lessines, Belgium) at 5.8 kcal/day (1, 2).

In the first animal study, mice received twice daily subcutaneous injections of either 150 mg/day D,L-3HB sodium salt or isocaloric/isovolumetric D-glucose as placebo. Pair-fed healthy mice served as controls. After 5 days, mice were anesthetized and sacrificed by cardiac puncture, plasma, liver and skeletal muscle were snap frozen.

In the second animal study, on day 2 or day 5, septic mice received a subcutaneous injection of 75 mg D,L-3HB-^13^C_4_ sodium salt (606030, Sigma Aldrich, St. Louis, MO, USA). As controls, full-fed mice received an equal tracer bolus injection. Two hours post-injection, mice were anaesthetized and sacrificed by cardiac puncture, plasma, liver and skeletal muscle were snap frozen.

***Mouse study 1– tissue and plasma analyses***

Plasma cholesterol was measured with the Amplex™ Red Cholesterol Assay Kit (Thermo Fisher Scientific, Waltham, MA, USA). Total and free cholesterol concentrations were measured with and without the addition of esterase, respectively. Cholesteryl ester concentration was calculated by subtracting the free from the total cholesterol concentration. Plasma mevalonate and ubiquinone-9 concentrations were measured with LC/MS by BioNotus (Niel, Belgium).

From muscle and liver tissue, messenger RNA was isolated, and cDNA was quantified in real-time as previously described (3). Commercial TaqMan® assays (Applied Biosystems, Carlsbad, CA, USA) were used for all gene expression analyses (Supplemental table 1). Data were normalized to *Rn18s* or *Hprt* and expressed as fold change of the mean of controls.

Citrate synthase and mitochondrial respiratory chain complex activities were measured with spectrophotometry at 30°C as described previously, with minor changes (4). Tibialis muscle (1:15 w/v) was homogenized in homogenization buffer (210 mM mannitol, 70 mM sucrose, 5 mM HEPES, 1 mM EGTA, pH 7.2) on ice with a Dounce homogenizer, followed by five freeze-thaw cycles in liquid nitrogen and an additional sonication step. Activities were calculated by subtracting the blank measurements from the total absorbance. Samples were measured in triplicate. Citrate synthase activity was measured in a buffer containing 50 mM potassium phosphates, pH 7.4 and 100 µM Ellman’s reagent. The reaction was started by adding the homogenate, 100 µM acetyl-CoA and 100 µM oxaloacetic acid, pH 7.2. For blanks, oxaloacetic acid was omitted. The production of the thionitrobenzoate anion was followed at 412 nm. Complex I activity was measured in a buffer containing 50 mM KPi pH 7.4, 50 µM NADH, 1 mM KCN, 10 µM antimycin A, 1 mg/ml BSA and 50 µM coenzyme Q1. The reaction was started after adding the homogenate. To the blank, complex I inhibitor 2.5 µM rotenone was added. NADH consumption was followed at 340 nm. To correct for protein content, muscle tissue samples were homogenized in a buffer containing 20 mM Tris-HCl, pH 7.6, 10% glycerol, 1% Nonidet P-40, 2 μg/ml aprotinin, 5 μg/ml leupeptin, 0.5 μg/ml pepstatin, 10 mM sodium orthovanadate, 34 μg/ml phenylmethylsulfonyl fluoride, 10 mM sodium pyrophosphate, 100 mM sodium fluoride, and 10 mM EDTA. The protein content was determined with Coomassie Protein Assay Reagent (Thermo Fisher Scientific) using a standard curve of BSA.

To quantify cholesterol tissue content, the extensor digitorum longus (EDL) muscle (1:40 w/v) was homogenized in 50% methanol + 0.5% acetic acid with ceramic beads in a Precellys tissue homogenizer (Bertin instruments, Montigny-le-Bretonneux, France). An equal volume of hexane was added to extract lipids. The phases were mixed by vortexing every 10 min during an hour. After centrifugation, the hexane phase was transferred to a glass tube and the water phase was extracted one more time with hexane, after which both hexane phases were combined. Part of the hexane phase (80 µl) was evaporated using a GeneVac EZ-2 (Genevac Limited, Ipswich, UK) after the addition of 2% Thesit. As previously described, Thesit enables more efficient and complete esterase action during enzymatic cellular cholesterol measurements in aqueous media, such as assay buffers (5). After dissolving evaporated lipid fractions in assay buffer (Amplex™ Red Cholesterol Assay Kit), total cholesterol was measured in duplo with the Amplex™ Red Cholesterol Assay Kit according to the manufacturer’s instructions. Protein content was determined as described above.

***Mouse study 2 – ^13^C-3HB uptake and metabolism***

Frozen tissue samples and plasma were homogenized in 80% methanol with ceramic beads in a Precellys tissue homogenizer for liver (Bertin instruments) or with a Dounce homogenizer for gastrocnemius muscle. After 5 min centrifugation at 20,000 g, the supernatant was transferred to a vial. Measurements were performed using a Dionex UltiMate 3000 LC System (Thermo Fisher Scientific) in-line connected to a Q-Exactive Orbitrap mass spectrometer (Thermo Fisher Scientific). A volume of 10 µl of the sample was injected on a C18 column (Aquility UPLC®HSS T3, 1.8 µm, 2.1 x 100 mm) with an ACQUITY UPLC HSS T3 VanGuard Pre-column (100 Å, 1.8 µm, 2.1 mm X 5 mm). The following gradient was performed by solvent A (H_2_O, 10 mM tributylamine, 15 mM acetic acid) and solvent B (100% methanol). Chromatographic separation was achieved with a flowrate of 0.25 ml/min and the following gradient elution profile: 0 min, 5% B; 2 min, 5% B; 7 min, 37% B; 14 min, 41% B; 26 min, 95% B; 30 min, 95% B; 31 min, 5% B; 40 min, 5% B. The column was placed at 40°C throughout the analysis. For tracer incorporation analysis in 3HB, citrate, fumarate, and mevalonate, the mass spectrometer operated in negative full scan mode between m/z 70 to 1050, using a spray voltage of 3.2 kV, capillary temperature of 320°C, sheath gas at 40.0, auxiliary gas at 15.0. For Full scan, AGC target was set at 3e6 using a resolution of 140,000, with a maximum IT of 512 ms. For each individual mouse, the fractional contribution of each labeled metabolite (indicating the fraction labeled metabolite of the total amount of metabolite) in each tissue was corrected for the fractional contribution of 3HB in plasma.

**Additional file 1: Table S1:** List of commercial TaqMan® assays

| ***Gene*** | ***Assay ID*** |
| --- | --- |
| ribosomal 18S (*Rn18s*) | Mm04277571_s1 |
| hypoxanthine guanine phosphoribosyl transferase (*Hprt*) | Mm03024075_m1 |
| acetoacetyl-CoA synthetase (*Aacs*) | Mm00513427_m1 |
| 3-hydroxy-3-methylglutaryl-CoA synthase 1 (*Hmgcs1*) | Mm01304569_m1 |
| 3-hydroxy-3-methylglutaryl-CoA reductase (*Hmgcr*) | Mm01282499_m1 |
| farnesyl diphosphate farnesyl transferase 1 (squalene synthase; *Fdft1*) | Mm01598574_g1 |
| sterol regulatory element binding factor 2 (*Srebf2*) | Mm01306292_m1 |
| ATP Binding cassette subfamily G member 5 (*Abcg5*) | Mm00446241_m1 |
| ATP Binding cassette subfamily G member 8 (*Abcg8*) | Mm00445980_m1 |
| ATP Binding cassette subfamily A member 1 (*Abca1*) | Mm00442646_m1 |
| ATP Binding cassette subfamily G member 1 (*Abcg1*) | Mm00437390_m1 |
| low density lipoprotein receptor (*Ldlr*) | Mm01177349_m1 |
| lipoprotein lipase (*Lpl*) | Mm00434764_m1 |
| scavenger receptor class B member 1 (*Scarb1*) | Mm00450234_m1 |
| acetyl-CoA acetyltransferase 1 (*Acat1*) | Mm00507463_m1 |
| neutral cholesterol ester hydrolase 1 (*Nceh1*) | Mm00626772_m1 |

**Additional file 1: Table S2:** *Multivariate linear regression analysis of factors determining day 3/LD serum cholesterol*

| Independent determinants of serum cholesterol day3/LD | | |
| --- | --- | --- |
| **R^2^=0.07 (p<0.001)** | **Estimate (95%CI)** | **P-value** |
| Statin therapy | -0.37 (-0.73 – -0.01) | **0.04** |
| Randomization to early-PN^B^ | -0.08 (-0.36 – 0.19) | 0.54 |
| Baseline risk factors |  |  |
| Age (per year added) | -0.01 (-0.02 – 0.01) | 0.8 |
| Female gender | 0.31 (0.03 – 0.60) | **0.02** |
| BMI (25-40 kg/m^2^) | 0.17 (-0.11 – 0.46) | 0.2 |
| NRS ≥5 | -0.32 (-0.65 – 0.01) | **0.05** |
| APACHE II (per unit added) | -0.07 (-0.11 – -0.04) | **<0.0001** |
| Presence of diabetes | 0.08 (-0.30 – 0.48) | 0.6 |
| Presence of malignancy | 0.07 (-0.26 – 0.41) | 0.6 |
| Pre-admission dialysis | 0.60 (-1.99 – 0.78) | 0.3 |
| Sepsis upon admission | 0.23 (-0.10 – 0.58) | 0.1 |
| Admission categories (compared with medical ICU) |  |  |
| Surgical ICU, emergency | 0.72 (0.19 – 1.25) | **0.007** |
| Surgical ICU, elective | -0.66 (-1.66 – 0.33) | 0.1 |
| Cardiac surgery | -0.45 (-1.05 – 0.14) | 0.1 |

BMI is body-mass index, or weight in kilograms divided by the square of the height in meters. APACHEII reflects scores on the Acute Physiology and Chronic Health Evaluation II (APACHE II) range from 0 to 71, with higher scores indicating a greater severity of illness. NRS reflects Nutritional Risk Screening (NRS) scores which range from 0 to 7, with higher scores indicating a higher risk of malnutrition.

**
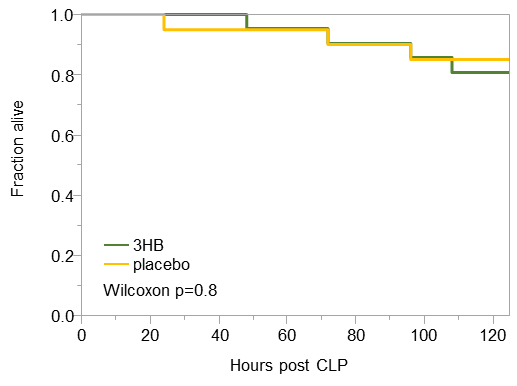
**

**Additional file 1: figure S1 – Survival analysis of 3HB vs. placebo treated septic mice.** Septic mice received parenteral nutrition supplemented with 3-hydroxybutyrate (3HB) or isocaloric glucose as placebo (placebo). [Orange: septic mice receiving placebo, n=17/20; green: septic mice receiving 3HB, n=17/21].

**References**

1. Derde S, Thiessen S, Goossens C, Dufour T, Van den Berghe G, and Langouche L. Use of a Central Venous Line for Fluids, Drugs and Nutrient Administration in a Mouse Model of Critical Illness. *Jove-Journal of Visualized Experiments.* 2017(123).

2. Goossens C, Weckx R, Derde S, Dufour T, Vander Perre S, Pauwels L, et al. Adipose tissue protects against sepsis-induced muscle weakness in mice: from lipolysis to ketones. *Crit Care.* 2019;23(1):236.

3. Langouche L, Marques MB, Ingels C, Gunst J, Derde S, Vander Perre S, et al. Critical illness induces alternative activation of M2 macrophages in adipose tissue. *Crit Care.* 2011;15(5):R245.

4. Thiessen SE, Derese I, Derde S, Dufour T, Pauwels L, Bekhuis Y, et al. The Role of Autophagy in Critical Illness-induced Liver Damage. *Sci Rep.* 2017;7(1):14150.

5. Van Veldhoven PP, Meyhi E, and Mannaerts GP. Enzymatic quantitation of cholesterol esters in lipid extracts. *Anal Biochem.* 1998;258(1):152-5.
